# Complete genome sequence of *Halomonas* sp. strain KFRI-KJS25024, isolated from fermented shrimp source (saeu-jeotgal) in Gang-Gyeong, Korea

**DOI:** 10.1128/mra.01078-25

**Published:** 2025-12-10

**Authors:** Myunglip Lee, Yukyoung Park, Sunghun Yi

**Affiliations:** 1 Fermentation Convergence Research Group, Korea Food Research Institute (KFRI), Wanju-gun, Jeollabuk-do, South Korea; Montana State University, Bozeman, Montana, USA

**Keywords:** *Halomonas* sp., KFRI-KJS25024, fermented shrimp source, *Halomonadaceae*

## Abstract

The complete genome of *Halomonas* sp. strain KFRI-KJS25024, isolated from fermented shrimp sauce (saeu-jeotgal), was obtained using PacBio HiFi and Illumina sequencing. The 3.38 Mb circular genome (64.6% GC) encodes 2,947 protein-coding sequences, 66 tRNAs, and 15 rRNAs, providing a valuable resource for comparative and functional genomics.

## ANNOUNCEMENT

The genus *Halomonas* (phylum *Pseudomonadota*, family *Halomonadaceae*) comprises moderately halophilic, gram-negative bacteria frequently isolated from saline environments and fermented seafoods. Some members have also been recovered from clinical settings, underscoring their ecological and applied relevance (1–10). Strain KFRI-KJS25024 was isolated from a traditional Korean fermented shrimp source (saeu-jeotgal) produced in Gang-gyeong, Korea. A 20 g sample was homogenized with 180 mL of sterile 0.85% (wt/vol) NaCl using a stomacher, serially diluted (10^3^ to 10^5^), and spread plated onto marine agar. Plates were incubated aerobically at 30°C for 72 hours. A single colony was purified, maintained on marine agar for routine use, and preserved at −80°C in glycerol stocks. The same isolate was used for genomic DNA extraction. Genomic DNA was extracted using the QIAamp DNA Mini Kit (QIAGEN) according to the manufacturer’s protocol. Whole-genome sequencing and annotation were performed using a hybrid approach combining Illumina NovaSeq X short-read libraries (150 bp) and PacBio Revio HiFi long-read libraries. Read quality was assessed with FastQC v.0.11.7, and Illumina reads were trimmed with Trimmomatic v.0.38 (11, 12). K-mer counting was performed using Jellyfish v.1.1.12 (13). PacBio HiFi long-read assembly was generated using Canu v.1.7 and refined through the Microbial Genome Analysis pipeline (SMRT Link v.13.1.0.221970). Sequencing was performed using HiFi circular consensus sequencing (CCS) chemistry on the PacBio Revio system. Raw subreads were processed with the CCS algorithm in SMRT Link v.13.1.0 to generate HiFi reads, and the final hybrid assembly was polished with Illumina reads using Pilon v.1.22 (14). Genome completeness was assessed with BUSCO v.5.1.3 (15). Default parameters were used except where otherwise stated. Annotation generated by PGAP was cross-validated with Prokka v.1.14.6, with orthology and functional assignments complemented by eggNOG v.4.5, and discrepancies were resolved in favor of PGAP results (16–18). Pairwise 16S identity and genome average nucleotide identity (ANI) analyses were obtained from the EZBioCloud platform (19, 20).

Illumina and PacBio HiFi sequencing generated 3.3 Gbp (21,625,902 paired-end reads) and 420.8 Mbp of data, achieving 98.39% completeness (BUSCO v.5.1.3, bacteria_odb10). Sequencing libraries were prepared using the TruSeq Nano DNA Library Prep Kit (Illumina) and the SMRTbell Express Template Prep Kit 3.0 (PacBio, USA), with size selection according to the manufacturer’s protocol. The hybrid assembly yielded a single circular chromosome of 3,375,639 bp with 64.6% GC content and an average sequencing depth of 124.6×. Genome annotation predicted 3,058 genes, including 2,947 protein-coding sequences, 66 tRNAs, 15 rRNAs, and 4 ncRNAs (Table 1). The genome of strain KFRI-KJS25024 encodes a PQQ-dependent sugar dehydrogenase, suggesting potential involvement in oxidative carbohydrate metabolism, as well as several universal stress protein genes. 16S rRNA gene analysis indicated that the closest relative was *Halomonas alimentaria* YKJ-16ᵀ (GenBank accession no. AF211860), showing 98.08% sequence similarity. However, the ANI between KFRI-KJS25024 and *H. alimentaria* (GenBank accession no. GCF_009902005.1) was only 85.49%, well below the 95% cutoff for species delineation (21). These results indicate that strain KFRI-KJS25024 represents a distinct lineage within the genus *Halomonas*. This genome provides a resource for studying halophilic adaptation and microbial diversity and may aid biotechnological applications of *Halomonas* in food and saline bioprocesses.

**TABLE 1:** Genome assembly and annotation statistics for *Halomonas* sp. KFRI-KJS25024

Feature	Value
Genome size (bp)	3,375,639
GC content (%)	64.6
Contigs	1 (circular)
Sequencing depth (×)	124.6
Protein-coding sequences	2,947
rRNA genes	15
tRNA genes	66
ncRNAs	4

## Data Availability

The complete genome sequence of *Halomonas* sp. strain KFRI-KJS25024 has been deposited in GenBank under accession number CP199872. The BioProject accession number is PRJNA1321417, and raw reads are available in the Sequence Read Archive under accession number SRS26431984.
